# A Comprehensive Survey of Abstractive Text Summarization Based on Deep Learning

**DOI:** 10.1155/2022/7132226

**Published:** 2022-08-01

**Authors:** Mengli Zhang, Gang Zhou, Wanting Yu, Ningbo Huang, Wenfen Liu

**Affiliations:** ^1^State Key Laboratory of Mathematical Engineering and Advanced Computing, Zhengzhou, China; ^2^Guilin University of Electronic Technology, Guilin, China

## Abstract

With the rapid development of the Internet, the massive amount of web textual data has grown exponentially, which has brought considerable challenges to downstream tasks, such as document management, text classification, and information retrieval. Automatic text summarization (ATS) is becoming an extremely important means to solve this problem. The  core of ATS is to mine the gist of the original text and automatically generate a concise and readable summary. Recently, to better balance and develop these two aspects, deep learning (DL)-based abstractive summarization models have been developed. At present, for ATS tasks, almost all state-of-the-art (SOTA) models are based on DL architecture. However, a comprehensive literature survey is still lacking in the field of DL-based abstractive text summarization. To fill this gap, this paper provides researchers with a comprehensive survey of DL-based abstractive summarization. We first give an overview of abstractive summarization and DL. Then, we summarize several typical frameworks of abstractive summarization. After that, we also give a comparison of several popular datasets that are commonly used for training, validation, and testing. We further analyze the performance of several typical abstractive summarization systems on common datasets. Finally, we highlight some open challenges in the abstractive summarization task and outline some future research trends. We hope that these explorations will provide researchers with new insights into DL-based abstractive summarization.

## 1. Introduction

In the digital era, cloud resources, such as webpages, blogs, news, user messages, and social network platform, have accumulated gigantic amounts of textual data, and they are increasing exponentially every day. In addition, various articles, books, novels, legal documents, scientific papers, biomedical documents, and other archives also contain rich textual content. As a result, information overload is becoming more and more serious. Almost every day, users must spend a lot of time browsing all kinds of cumbersome texts and filtering out redundant information, which dramatically reduces their efficiency [1–11]. Therefore, how to quickly locate the information needed from the text resources, then summarize and compress it, has become an urgent and fundamental problem to be solved. Manual summarization requires browsing all the content and then summarizing, which is very expensive and easily lost in massive data. Automatic text summarization (ATS) provides an effective way to solve this problem [12–21].

ATS aims to automatically generate a concise and readable summary containing the core contents of the input text. It is becoming more and more important for solving how to obtain required information quickly, reliably, and efficiently. Due to the complexity of input text, ATS has become one of the most challenging tasks in the field of natural language processing (NLP) [22–34]. As early as 1958, Luhn [35] began the study of ATS. They proposed to automatically extract the summaries from magazine articles and technical papers. In 1995, Maybury [36] constructed a system that can select key information from an event database and defined a high-quality summary as the most essential content extracted from the input document. In 2002, Radev et al. [37] also defined the summary as a combination of sentences generated from multiple (or one) input documents, which contains the core contents of input documents. They emphasized that the length of the generated summary is no more than half of the input or even less. These previous descriptions capture many essential characteristics of the ATS tasks, that is, the summaries should cover the core contents of the input document and be concise.

Generally, there are two prominent summarization systems based on the way the summaries are generated: extractive summarization [38–41] and abstractive summarization (ABS) [42–53]. Extractive systems directly extract sentences or phrases from the original document to form a summary, including graph-based methods (e.g., LexRank [54]), centrality-based methods (e.g., Centroid [55]), and corpus-based methods (e.g., TsSum [56]). Abstractive systems need to first understand the semantics of the text, and then employ the algorithm of natural language generation (NLG) to generate a more concise summary using paraphrase, synonymous substitution, sentence compression, etc. Therefore, compared with extractive summarization, the concept of ABS is closer to the process of handwritten summaries [57]. However, for a long time, due to the limitations of traditional methods in textual representation, understanding, and generation ability, the development of ABS is slow, and the effect is also worse than that of extractive summarization [58].

Recently, with the continuous improvement of neural network theory and technology, deep learning (DL) has become one of the most effective and promising methods, and has achieved SOTA effect on a lot of tasks [59–66], such as image processing, computer vision (CV), NLG, NLP, etc. In 2015, Rush et al. [67] first transferred deep learning technology to ABS. They constructed an ABS model based on encoder-decoder architecture. After that, various improved ABS models were developed, all of which were deep neural networks built under the encoder-decoder architecture. To this day, the research community's enthusiasm for DL-based ABS has been unabated, and many excellent methods have emerged. Moreover, the results of DL-based ABS are still constantly being refreshed.

As more and more researchers devote themselves to ABS research, an overview is urgently needed to help them quickly and comprehensively understand the achievements and challenges in this field. In this work, we aim to fill this gap.  Table 1 shows the main directions of our efforts in this paper. To this end, we focus on DL-based ABS tasks and review their development process. We also summarize some popular basic frameworks and improved methods. Then, we analyze the performance of the existing models and objectively describe their advantages and shortcomings. Moreover, we compare their results on large-scale public datasets using some popular evaluation metrics. Finally, we highlight some open challenges in the ABS task and outline some future research trends. Specifically, compared with some similar work, we further expand the following four aspects: (1) From the perspective of methodology, we classify some popular models in recent years; (2) we define a new type of model, which deals with the problem of factual errors, and conduct in-depth analysis on them; (3) we summarize the ROUGE scores of all SOTA models in the past 5 years, and visually show the development process of summarization technology based on deep learning; (4) and we discuss the possible hotspots of future research from the perspective of application.

The main contributions of our work are as follows:

- We provide a systematic overview of the DL-based ABS approaches and detail several popular frameworks under the encoder-decoder architecture.
- We classify the DL-based ABS, elaborate the framework of each class, and analyze the advantages and disadvantages.
- We provide a comprehensive overview of commonly used datasets and evaluation metrics in the ABS tasks. We also report the performance analysis results of different models on large-scale datasets, which should be helpful for researchers to choose a suitable framework and model according to their own needs.
- We discuss several directions worth studying and provide some new perspectives and inspirations for future research and application of ABS.

## 2. Preliminaries

### 2.1. Problem Formulation

ABS is an intersecting task of natural language understanding (NLU) and NLG. It needs to perform semantic analysis on the input document first, and then employ some NLG techniques to generate short summary sentences. Specifically, given one or more input documents **D** consisting of many tokens (*w*_1_, *w*_2_, ⋯, *w*_*n*_), ABS aims to generate a shorter description **Y**=(*y*_1_, *y*_2_, ⋯, *y*_*m*_) that captures the gist of **D**, and usually *m* < n/2. Among them, all tokens come from a pre-defined fixed vocabulary *𝒱*.

**Figure 1:**
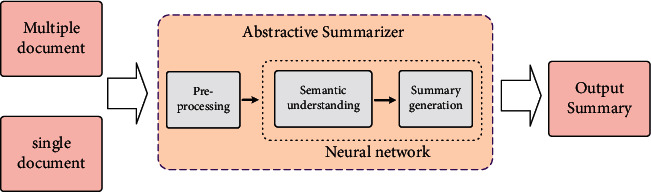
A general architecture of DL-based ABS. It is mainly composed of three steps: preprocessing, semantic understanding, and summary generation.

Figure 1 depicts a general architecture of DL-based ABS, which is mainly composed of three steps: preprocessing, semantic understanding, and summary generation. In the preprocessing step, some linguistic technologies are mainly used to structure the input text, such as sentence segmentation, word tokenization, and stop-word removal, etc. In the semantic understanding step, a neural network is constructed to recognize and represent the deep semantics of the input text. This step occurs in the vector space, and finally generates a fusion vector for the next step. In the summary generation step, the generator makes appropriate adjustments to the fusion vector provided in the previous step, and then maps the vector space representation to the vocabulary to generate summary words.

### 2.2. Deep Neural Networks

Deep neural networks (DNNs) are the foundation of deep learning, which use sophisticated mathematical methods to train various models. It contains many hidden layers, so it is sometimes called a multi-layer perceptron (MLP). In this section, we introduce several DNNs commonly used in ABS, including recurrent neural networks (RNN), convolutional neural network (CNN), and graph neural network (GNN).

#### 2.2.1. Recurrent Neural Network

**Figure 2:**
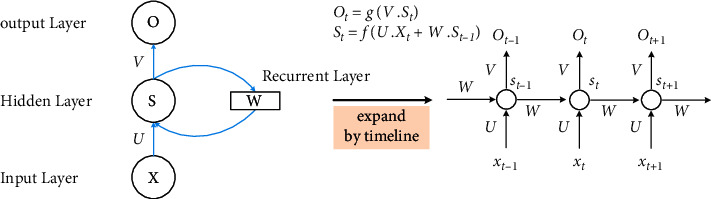
RNN timeline expansion diagram.

The proposal of RNN is based on an intuitive understanding that “human's cognition is based on experience and memory.” In RNN, there is a sequential relationship within the sequence, and adjacent items depend on each other. The network predicts the output of the next time step by combining the characteristics of the input at the previous and the current timestep. Specifically, the hidden layer nodes of RNN are connected to each other. The hidden layer input is composed of the output of the input layer and the previous hidden layer. The structure of RNN is shown in Figure 2 [68]. Given an input sequence **D**=(*w*_1_, *w*_2_, ⋯, *w*_|**D**|_), where *w*_*t*_ (*t* ≤ *| ***D***|*) denotes the input token at timestep *t*, RNN can output the vector representation of **D**, which is **h**=(*h*_1_, *h*_2_, ⋯, *h*_|*X*|_).

RNN is very effective in processing sequential data. It can mine temporal and semantic information in data. Therefore, the RNN-based DL models have made breakthroughs in solving some challenging problems in NLP, such as information extraction (IE), recommender system, machine translation, text summarization, and timing analysis. However, when the sequence is too long, RNN begins to appear gradient explosion and disappear. To alleviate this problem, Cheng et al. [68] constructed a novel neural network called Long Short Term Memory (LSTM). Different from RNN, LSTM selectively stores information through the input, forget, and output gates, which largely solves the problem of long-term dependencies. On the basis of LSTM, Cho et al. [69] further simplified the network structure. They used an update gate to replace the input and forget gates and proposed a novel Gate Recurrent Unit (GRU). Furthermore, by increasing the flow of information from back to front, the bidirectional RNNs are proposed, denoted as: Bi-RNN, Bi-LSTM, and Bi-GRU.

#### 2.2.2. Convolutional Neural Network

**Figure 3:**
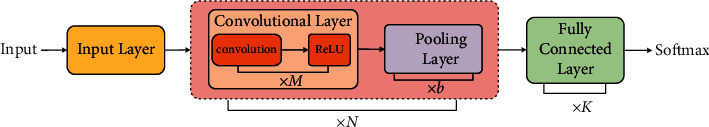
Framework of convolutional neural networks.

CNN [70] is a deep feedforward neural network composed of many convolution operations. The neurons in CNN are arranged in three dimensions, that is, depth, width, and height. Neurons in different layers are no longer fully connected but connected between a small area. The most notable features of CNN are equivariant representations, sparse interactions, and parameter sharing, providing a way for neural network models to handle inputs of varying sizes. The basic CNN consists of three structures: convolution, activation, and pooling. CNN employs the convolution kernel to extract features from the data object, and uses maximum pooling on the extracted features at intervals, which can obtain different levels of features from simple to complex. The convolution filter and pooling operations can not only identify the important characteristics of the input matrix but also greatly simplify the complexity and reduce the parameters. One of the convolution blocks is composed of consecutive *M* convolutional layers and *b* pooling layers. In a CNN, *N* convolutional blocks can be stacked consecutively, and *K* fully connected layers are connected at the end. Generally, *M* is set to 2–5, *b* is 0 or 1, *N* is 1–100 or more, and *K* is 0–2. The structure of a commonly used typical CNN is shown in Figure 3. As the core technology of CV, CNN plays an essential role in the image field. Classical CNN includes Lenet, Alexnet, GoogleNet, VGG, etc. In recent years, CNN has been expanding in face recognition, machine translation, motion analysis, and NLP, and has achieved good results.

#### 2.2.3. Graph Neural Network

**Figure 4:**
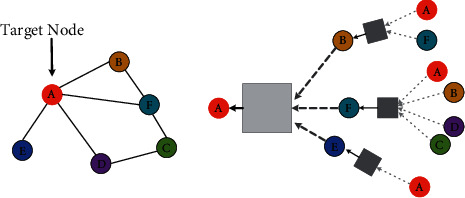
The  schematic diagram of GNN. The  basic idea of GNN is to embed nodes according to the local neighbourhoods.

GNN [71] is a neural network that specializes in processing graph data. A basic idea of GNN is to embed nodes according to the local neighbourhoods. Intuitively speaking, the characteristics of each node and the nodes connected to it are aggregated through a neural network. The schematic diagram of GNN is shown in Figure 4 [71]. The embedding of node *v* in the *k*-th layer is calculated as follows [71]:

(1) $$\begin{array}{c} h_{v}^{0}=x_{v}, \end{array}$$

(2) $$\begin{array}{c} h_{v}^{k}=\sigma \left({}_{Wk}\sum_{u\in N\left(v\right)}\frac{h_{u}^{k-1}}{\left|N\left(v\right)\right|}+B_{k}h_{v}^{k-1}\right),\;\forall k>0, \end{array}$$

where **h**_*v*_^0^ is the embedding of node *v* at *0*-th layer, **h**_*v*_^*k*^ is the embedding of node *v* at *k*-th layer, and *𝒩*(*v*) is the set of neighbour nodes of *v*. At present, there are mainly four types of GNN, namely, graph convolution networks (GCNs), graph attention networks (GANs), gated graph neural network (GGNN), and graph generative network (GGN).

## 3. Methodologies

In this section, we review and summarize the development of ABS from the perspective of methodology.

### 3.1. Seq2Seq Framework

**Figure 5:**
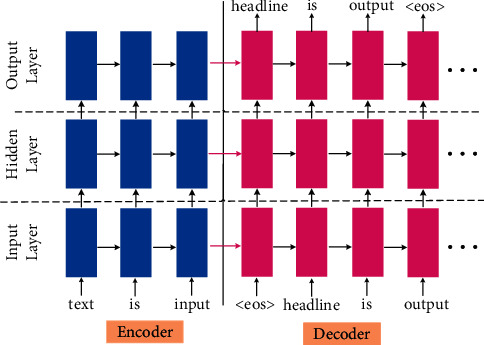
The basic encoder-decoder framework. It consists of input layer, hidden layer, and output layer.

The Seq2seq (sequence-to-sequence) framework, also known as the encoder-decoder framework, is widely regarded as the most efficient method in converting text from one form to another, such as speech recognition, question answering system, machine translation, etc. These models employ an encoder to identify, understand, and parse the input sequence, and use the high-dimensional dense feature vector to characterize it. Then, on the decoder side, the feature vectors of the input items are used to generate the output items gradually.  Figure 5 shows the basic encoder-decoder framework. The encoder-decoder framework is the most basic and core framework of DL-based ABS models. And, the encoder and decoder are constructed using various neural networks. A large number of research results are put forward based on encoder-decoder architecture [22–24], which makes the performance of the ABS models continuously improved.

### 3.2. Encoder-Decoder Systems with Basic Attention Mechanism

In 2015, Rush et al. [67] applied the encoder-decoder framework to the ABS for the first time. They proposed a novel ABS model with an attention mechanism. The model is mainly composed of the feed-forward neural language model (FFNLM), which is a parameterized neural network. The most significant advantage of their system is the use of a more powerful attention-based encoder (vs. Bag-of-Words encoder) and a beam search strategy [72] (vs. greedy decoding) to generate summaries.

**Figure 6:**
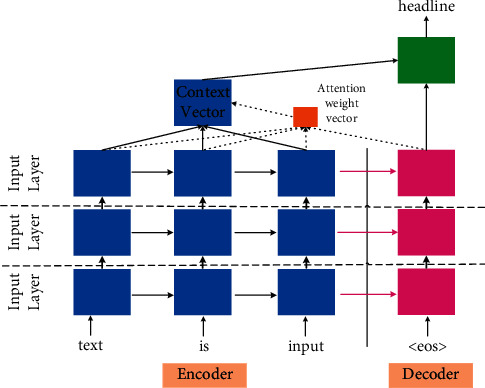
The basic encoder-decoder framework with attention mechanisms. The attention mechanism enables the decoder to interact with the input during the decoding process.

After that, Chopra et al. [73] further proposed a convolutional RNN model for ABS, which is an extension of the method proposed by Rush et al. [67]. The encoder of their model adopts a convolutional attention mechanism to ensure that the decoder aligns with the corresponding input token at each decoding time step, thus providing an adjustment function for the generation process. In addition, they also provided two optional networks for the decoder: Vanilla RNN and LSTM. The encoder-decoder framework with an attention mechanism is shown in Figure 6 [73]. The attention-based context vector is calculated as in equations (3)–(5) [73]:

(3) $$\begin{array}{c} e_{i}^{t}=v^{T}\tanh\left(W_{a}h_{i}+{}_{Wb}s_{t}+b_{attn}\right), \end{array}$$

(4) $$\begin{array}{c} \alpha_{i}^{t}={\text{softmax}}_{i}=\frac{\exp\left(e_{i}^{t}\right)}{\sum_{i=1}^{n}\exp\left(e_{i}^{t}\right)}, \end{array}$$

(5) $$\begin{array}{c} c_{t}=\sum_{i}\alpha_{i}^{t}h_{i}, \end{array}$$

where *α*_*i*_^*t*^ is the attention weight, which denotes the attention paid to the *i*-th token in the input when generating the *t*-th summary token. _**W***a*_ and _**W***b*_ are trainable parameters, *s*_*t*_ is the hidden layer state of the decoder at time *t*. Finally, the probability distribution at time step *t* is calculated as follows [73]:

(6) $$\begin{array}{c} p\left(w\right)=\text{softmax }\left({}_{Wo}s_{t}+V_{o}c_{t}+b_{gen}\right). \end{array}$$

Lopyrev et al. [74] tested two different attention mechanisms in the news headlines generation task. The first one is the same as the dot mechanism in Figure 5, and they called it *complex* attention. The second one is a slight variation of the dot mechanism consisting of some neurons used to calculate the attention weights, which has specific advantages when further exploring the functions of the network, and they called it *simple* attention. Their experiments showed that the *simple* attention mechanism performed better. Chen et al. [75] utilized the distraction-based Bi-GRU to model input document. In order to better model the overall document representation, they focused on specific regions and contents of the input text, while also distracting them from traversing between different contents of the input text. Their work is the early application of the coverage mechanism in ABS.

However, because RNN is difficult to control during the generation process, the basic encoder-decoder architecture still has some critical problems in ABS, such as generating out-of-vocabulary (OOV) words, modeling keywords, and capturing the hierarchical structure of words to sentences. To alleviate these problems, Nallapati et al. [76] further extended the basic encoder-decoder model. They constructed a feature-rich encoder, which uses an embedding vector for the Part-of-Speech (POS), named Entity Recognition (NER) tags, and discretized TF and IDF values, respectively. Then, these values are connected with word-based embedding values as the encoder input. The feature-rich-encoder can capture the key concepts and entities in the input document. They also employed a switching generator-pointer to model rare/unseen words in the input document, which alleviates the problem of generating OOV words. Moreover, they also introduced hierarchical attention to jointly model key sentences and keywords in the key sentences.

Furthermore, when processing longer documents (usually more than 1000 tokens), neural network-based models often generate repeated words and phrases, and even inconsistent phrases. To alleviate these problems, Paulus et al. [77] adopted the intra-attention method, which can pay attention to the specific area of input tokens and continuously generate output separately. At each decoding step, in addition to the decoder's hidden state and the previously generated tokens, their model also employs an intra-temporal attention function to pay attention to the specific area of input text. Thus, the intra-temporal attention can prevent the model from repeatedly paying attention to the same part in the original document at different decoding timesteps. To solve the problem of generating repeated phrases based on encoder hidden states, they further proposed to utilize intra-decoder attention to incorporate more information about the previously generated tokens into the decoder. At the current decoding time step, considering the tokens that have been generated allows the encoder-decoder model to make a more holistic decision, which can effectively avoid generating duplicate tokens, even if these tokens are generated many steps away.

### 3.3. Hierarchical Encoder-Decoder Models

**Figure 7:**
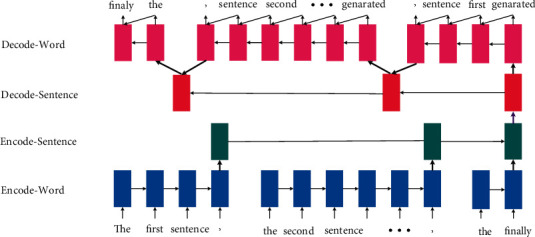
The basic hierarchical encoder-decoder architecture. It is mainly divided into sentence level and word level. The word level processes each word token, and the sentence level processes each sentence.

When the input is a lengthy document, the basic single-layer encoder-decoder architecture cannot fully capture the relationship between the contexts when encoding the document, which leads to the problem of long-distance dependence. Researchers found that long documents naturally have a hierarchical structure, that is, documents are composed of multiple long sentences (sentence level), and long sentences are composed of multiple words (sentence level). Inspired by this, researchers constructed a hierarchical encoder-decoder architecture. The hierarchical encoder-decoder architecture can significantly reduce long dependency problems. The basic framework of the hierarchical encoder-decoder ABS is shown in Figure 7.

Hierarchical neural models have shown strong performance in document-based language models (LM) [78] and some document classification [79] tasks. In 2015, Li et al. [80] proposed a basic hierarchical ABS model, and Jadhav and Rajan [81] further extended their model. And the summaries generated by their method are significantly better than similar methods in terms of informativity and readability. Inspired by the graph-based NLP models, Tan et al. [82] proposed a novel graph-based attention mechanism in the hierarchical encoder-decoder framework. They employed a word encoder to encode words, used a sentence encoder to encode short sentences, and utilized the hidden state of the sentences to construct a hidden state graph. The hierarchical attention value of the sentence is calculated from a hidden state graph.

Although the above hierarchical encoder-decoder model is designed based on the sentence-word hierarchy, it fails to capture the global structural characteristics of the document. In 2018, Li et al. [83] used the structural information of multi-sentence summaries and documents to enhance the performance of ABS models. In order to mine the information compression and information coverage properties, they proposed to model *structural-compression* and *structural-coverage* regularization during summary generation. They utilized sentence-level attention distributions to calculate the score of the *structural-compression*, as follows [83]:

(7) $$\begin{array}{c} strCom\left(\alpha_{t}\right)=1-\frac{1}{\log\;N}\sum_{i=1}^{N}\alpha_{t}^{i}\log\alpha_{t}^{i}, \end{array}$$

where *α*_*t*_^*i*^ is the sentence-level attention distribution. The *structural-coverage* of the summary is calculated as follows [83]:

(8) $$\begin{array}{c} strCov\left(\alpha_{t}\right)=1-\sum_{i}\min\left(\alpha_{t}^{i},\sum_{t^{\prime }=1}^{t-1}\alpha_{t}^{i}\log\alpha_{t^{\prime }}^{i}\right), \end{array}$$

which is used to encourage different summary sentences to concentrate on different source sentences in generating summary sentences. Their method achieved the SOTA results at the time.

Hsu et al. [84] found that the extractive summarization can get a high rouge score using sentence-level attention, but it is not easy to read. In addition, a more complex ABS model can obtain word-level dynamic attention, thereby generating more readable sentences. Inspired by this, they use sentence-level attention to adjust the attention assigned to each token, reducing the probability of tokens in sentences with less attention being selected. The updated word attention is calculated as follows [84]:

(9) $$\begin{array}{c} {\hat{\alpha }}_{m}^{t}=\frac{\alpha_{m}^{t}\times \beta_{n\left(m\right)}}{\sum_{m}\alpha_{m}^{t}\times \beta_{n\left(m\right)}}, \end{array}$$

where *α*_*m*_^*t*^ is word-level attention, *β*_*n*(*m*)_ is sentence-level attention. Moreover, they also proposed a novel inconsistency loss function to penalize the different attention between two different layers.

### 3.4. CNN-Based Encoder-Decoder Models

Unlike RNN that directly processes time-series data, CNN uses convolution kernels to extract features from data objects, which are often used in image-related tasks [85]. But after the text is represented by a distributed vector, each token is a matrix in the vector space. Then CNN can be used to perform convolution operations in text-related tasks [86]. In 2016, Facebook AI Research (FAIR) used CNN to build an encoder under the encoder-decoder architecture for the first time, and achieved SOTA results in machine translation tasks [87].

**Figure 8:**
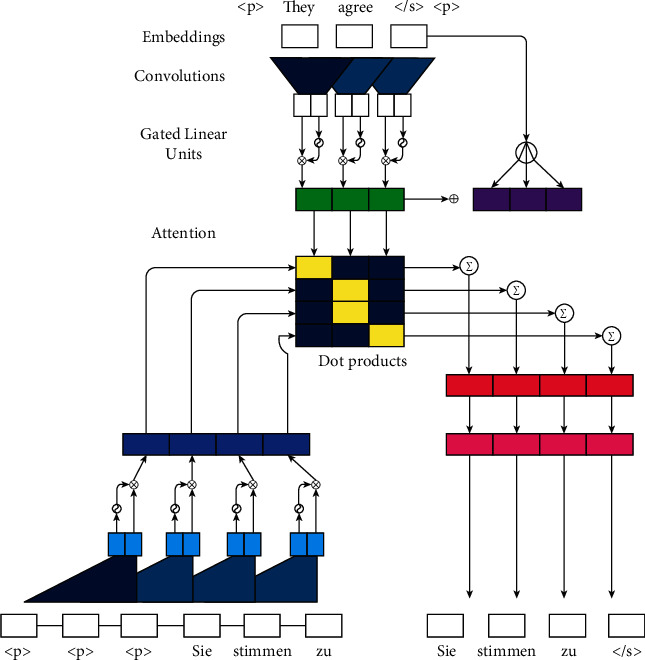
The CNN-based ABS model. It is the most representative ABS model based entirely on CNN.

In 2017, Gehring et al. [88] proposed a model ConvS2S and its encoder and decoder both use CNN, which is the most representative ABS model based entirely on CNN. The overall architecture of the model is shown in Figure 8 [88]. In their model, in addition to receiving the word embedding, the input layer also adds a position vector for each input token. Then, the word and position embeddings are concatenated to form the final embeddings of the word, which enables the CNN-based models to perceive the word order like RNN and use the convolution module to convolution and nonlinear transformation of the embedding. In addition, to alleviate the problem of gradient disappearance and explosion, they introduced residual connections between layers. Their model achieves similar results to the RNN-based models on DUC-2004 and Gigaword datasets, and the training speed is greatly improved.

Fan et al. [89] proposed a model that can specify the length, style, and entities of the summary, and other high-level attributes, which can control the shape of the generated summary and meet the needs of user customization. The encoder and decoder of their model are constructed by CNN. Inspired by Gehring et al. [88], they extended the intra-attention [87] to a multi-hop intra-attention. They also employed the self-attention mechanism on the decoder side to use the previous decoding information. To control the length of the generated summary, they first used the discrete bins to quantize summary length. Then, they extended the input vocabulary with special word types and used a marker to indicate the length of the ground-truth summary during training.

Narayan et al. [90] constructed an extreme ABS system that aims to generate a one-sentence title for answering the question “What is the article about?” Their model is a topic-conditioned architecture, and the encoder and decoder are both built on CNN. The convolutional encoder associates each token with a topic embedding to capture whether it represents the salient information of the document, while the decoder controls the prediction of each token. Specifically, they employed the LDA topic model [91] to obtain the topic embeddings of words and documents, which is the additional input of the encoder and decoder.

In sequence modeling, because the convolutional layer can only generate fixed-size context vectors, CNN-based ABS models cannot directly process variable-length sequence samples. However, the superposition of convolutional layers can increase the context representation, forming a hierarchical structure. The elements in the sequence can be calculated in parallel between layers, and the long-distance dependence problem between elements can be solved under a shorter path. Therefore, the training of the ABS model based on CNN is more efficient than RNN. However, compared with the chain structure of RNN, the hierarchical structure of CNN makes the adjustment of parameters greatly increase, which dramatically increases the cost of parameter adjustment when the model is trained on a large dataset.

### 3.5. Methods for Tackling OOV Words and Repetition Problems

For ABS systems, OOV words and repetition problems are one of the most important factors affecting model performance, and they are also the most common problems. Based on the statistics of the generated summaries, the researchers found that almost all OOV words can be found from the input document, and they are low-frequency words. Therefore, the researchers proposed that when generating the summary token, the model should be able to find and copy low-frequency words from the input document. In addition, to alleviate the problem of generating repeated words or phrases, the tokens that have been generated previously should be penalized (reduce the score) during the generation process to avoid generating duplicate tokens.

**Figure 9:**
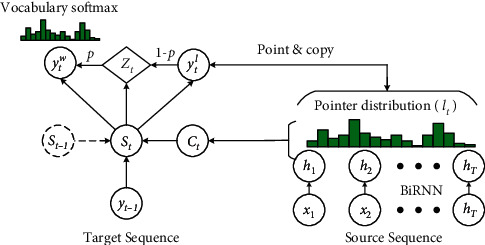
The framework of the pointer softmax. It utilizes two softmax layers to predict the next generated words: one softmax to predict the location of the word in the source sentence and copy it as output, and the other to predict the word in the shortlist vocabulary.

Gulcehre et al. [92] constructed a model to use an attention-based pointing mechanism to process rare and unseen words (OOV words). Their model employed two softmax layers to predict the next generated words: one softmax to predict the location of the word in the source sentence and copy it as output, and the other to predict the word in the shortlist vocabulary. In each prediction process, they use Multilayer Perceptron (MLP) to decide which softmax to use to generate words. At the same time, a large vocabulary trick (LVT) [93] is introduced, which reduces the size of the softmax layer in the decoder side and makes the decoding process more efficient. Their inspiration comes from a common human psychology: when people do not understand an entity's name, they tend to make guesses based on context and background. Their method significantly alleviates the problem of generating OOV words. The framework of the pointer softmax is shown in Figure 9 [92].

Gu et al. [94] proposed a new ABS model (CopyNet) based on the encoder-decoder framework, incorporating the copying mechanism into the decoding process. The CopyNet model can well combine the regular word generation method in the decoder with a new copy mechanism, which can select words and phrases in the input document and place them in the appropriate positions of the generated summary. Particularly, they conducted experiments on both synthetic and real datasets, and the results confirmed the effectiveness of their models in alleviating the OOV word problem.

**Figure 10:**
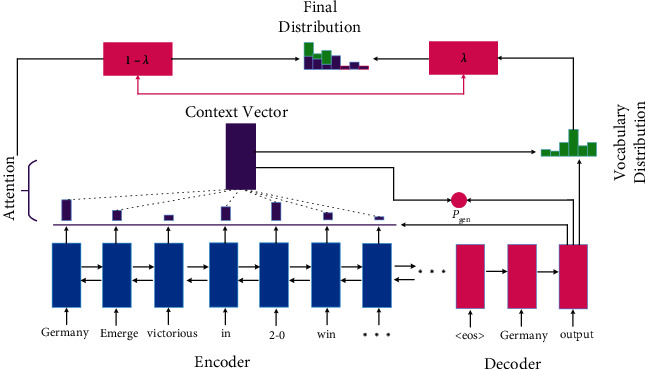
The framework of the PG model. It utilizes a pointer to copy words from the input document, which helps to accurately reproduce the information while retaining the ability to generate new tokens through the generator.

Furthermore, See et al. [95] proposed a more comprehensive ABS model with a point-generator (PG) network. The PG employs a pointer to copy words from the input document, which helps to accurately reproduce the information while retaining the ability to generate new tokens through the generator. In addition, to alleviate the problem of generating repeated words and phrases, they proposed a coverage mechanism to track what has been generated and punish them. Compared with the methods of Gulcehre [93] et al. and Nallapati et al. [76], PG is considerably different, with two main aspects: (1) The pointer of PG can freely select the words to be copied, while the pointers of the other two methods are only activated when processing OOV words or named entities; (2) The final generating distribution of PG is a combination of pointer distribution and vocabulary distribution, while the distribution of the other two models is independent. The framework of the PG model is shown in Figure 10 [95].

The PG significantly alleviates the problem of generating OOV words and repetition, but it is still limited by the following two problems: (1) The pointer can only copy exact words, ignoring possible distortions or abstractions, which limits its ability to capture a latent potential alignment; (2) The hard copy mechanism allows the model to have a strong copy orientation, which will cause most sentences to be generated by simply copying the source input. Based on this, Shen et al. [96] proposed a generalized pointer generator (GPG) to enhance potential alignment. Their model allows re-editing the word pointed to by the pointer instead of a simple hard copy and performing the editing by converting the pointed word embedding into a target space with a learned relation embedding. Compared with a hard copy in PG, GPG can capture more abundant potential alignments, which contributes to the controllability and interpretability of the ABS model.

### 3.6. Methods for Tackling Factual Errors Problems

For the ABS system, it is necessary to first understand the entire input document, and then generate a summary. This process inevitably involves tailoring, modifying, reorganizing, and fusing the input text, which makes the entire system uncontrollable and generates fake information. Some literature has studied the factual errors problems in ABS models [97–99], and they concluded that nearly 30% of summaries generated using ABS systems did not match the facts described in the original documents. Therefore, to enhance the usability of the ABS models, it is necessary to keep the summary consistent with the factual descriptions in the original text.

**Figure 11:**
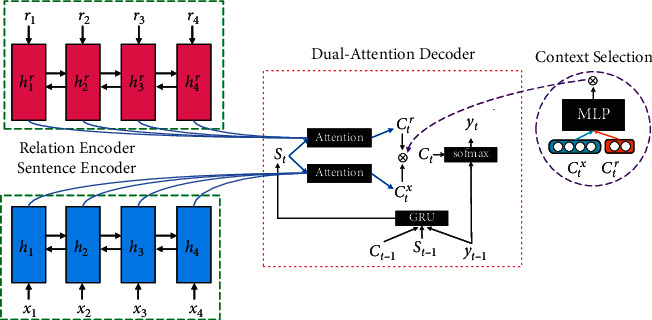
The overall framework of the FTSum model. It is a dual-attention encoder-decoder model.

In 2017, Cao et al. [100] proposed a dual-attention encoder-decoder model (FTSum) to enhance the factual correctness of their system. They first leveraged the Open Information Extraction (OpenIE) tool [101] to extract triples from the input as the fact descriptions of input text, then used a relational encoder to encode the fact descriptions. During decoding, they utilized the embedding of the fact descriptions and the original text to calculate the final attention. The new attention allows the model to pay more attention to the fact descriptions in the original text to avoid generating fake facts. The overall framework of the FTSum model is shown in Figure 11 [100].

**Figure 12:**
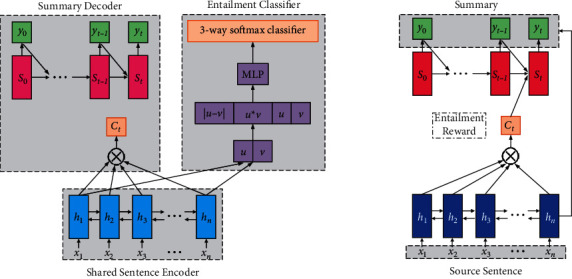
The overall framework of the Entailment-aware encoder-decoder model. It uses the attention-based encoder-decoder framework as the infrastructure, and then shares the encoder with the entailment recognition system.

Li et al. [102] adopted a multi-task learning strategy to introduce textual entailment [103] in the ABS task. Specifically, their model uses the attention-based encoder-decoder framework as the infrastructure, and then shares the encoder with the entailment recognition system, that is, uses the encoder in the ABS model and a softmax layer to construct an entailment relationship classifier trained on the NLI dataset. This enables the encoder not only to grasp the essence of the source document but also to be aware of the entailment relationship. Moreover, when decoding, they modified the loss function to reward the entailment degree of the generated summary and employed a Reward Augmented Maximum Likelihood (RAML) [104] to train the model, so that the decoder is also entailment aware. The overall framework of the model is shown in Figure 12 [102].

**Figure 13:**
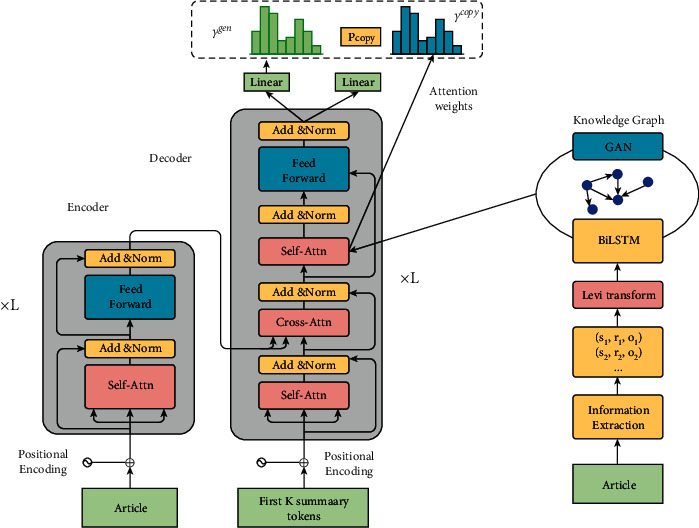
The overall framework of the FASum model. Its encoder and decoder are stacked by Transformer blocks.

Zhu et al. [105] proposed a Transformer-based encoder-decoder model (FASum), the encoder and decoder are stacked by Transformer blocks. They used open-source OpenIE [101] tool to extract entity relationship information from the original input text. The extracted knowledge is represented by a set of triples, where each triple is composed of a subject, an object, and a relation. For each triple (subject, relation, object), they regarded subject, relation, and object as three different nodes, and then connected two undirected edges *subject-relation* and *relation-object*. In this way, by constructing edges for all the triples, an undirected graph can be obtained, which is the knowledge graph of the input document. Then, the graph attention neural network [106] is used to extract the feature of each node on the knowledge graph, and this feature is used as the representation of the node. Finally, by constructing a cross-attention layer on the decoder side, the information of the knowledge graph is integrated into the decoding process to control the generation of the summary. The overall framework of the FASum model is shown in Figure 13 [105].

**Figure 14:**
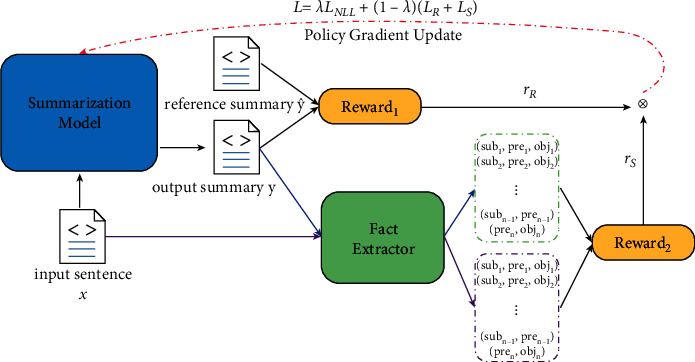
The overall framework of the FAR-ASS model.

Zhang et al. [107] proposed a fact-aware reinforced ABS model (FAR-ASS). They also employed the OpenIE and dependency parser tools to extract fact descriptions of the input document. Then, they elaborately designed a fact correctness evaluation algorithm, which can calculate the factual correctness score of generated summaries after comprehensively considering the fact correctness and redundancy. In the training phase, they adopted a reinforcement learning strategy based on fact correctness scores to train the summarization model. The overall framework of the FAR-ASS model is shown in Figure 14 [107].

## 4. Datasets

In this section, we provide an overview about the well-known and standard datasets, including: Document Understanding Conference (DUC) datasets, Text Analysis Conference (*TAC*) datasets, *CNN/DailyMail*, *Gigaword*, New York Times (*NYT*), *Newsroom*, Large-scale Chinese Short Text Summarization (*LCSTS*), etc.

### 4.1. DUC/TAC

The *DUC* datasets have become the most widely used and common datasets in the ABS research field. These datasets are collected and released by the National Institute of Standards and Technology (NIST). Every year, they provide a new set of English documents for researchers to evaluate their summarization system. Since 2008, *DUC* datasets have become a summarization track of *TAC*. Each item in *DUC/TAC* contains a news document and its corresponding ground-truth summaries. These summaries consist of three forms, including: (1) manually generated summaries, (2) summaries that are automatically generated as baselines, and (3) summaries that are automatically generated by challenge participants systems. And the *DUC/TAC* datasets are usually used as the testing set to evaluate the performance of the ABS model, because they contain a small amount of data, which is not enough to train neural network models [108]. The statistics of *DUC/TAC* datasets are shown in Table 2.

### 4.2. CNN/Daily Mail

The *CNN/Daily Mail* dataset [109] is used in passage-based question answering systems and has become the most widely used benchmark dataset in the field of abstractive text summarization. In 2016, Nallapati et al. [76] modified the original corpus to contain multi-sentence summaries, which is more used in the field of abstractive text summarization. The statistics of the *CNN/Daily Mail* dataset are shown in Table 3. Currently, there are two most popular versions of the *CNN/Daily Mail* dataset, as follows:

1. *Anonymized Version* [76, 79]. For each document-summary pair, the named entity in it is manually replaced with a unique identifier. For example, the entity *The United States* is replaced with the identifier *@entity7.*
2. *Nonanonymized Version* [95]. The original document-summary pair contains entity information.

### 4.3. Gigaword

The *Gigaword* dataset consists of about 10 million English news documents from different news agencies. In 2015, to train their ABS model, Rush et al. [67] preprocessed the original *Gigaword* dataset. They lowercase all English words, replace all digits with special characters, replace all undisplayable characters with UNK, and delete all duplicate phrases and sentences. Finally, the *Gigaword* dataset for ABS contains about 3.8 million training pairs, 189,000 validation pairs, and 2,000 commonly used testing pairs. Therefore, the *Gigaword* dataset is sufficient to train and test neural network models. However, since only the first sentence of the document is used as the ground-truth summary, the text summarization task on the *Gigaword* dataset is also called the headline (title) generation task. The statistics of the *Gigaword* dataset are shown in Table 3.

### 4.4. NYT

The *NYT* dataset comprises millions of articles in the New York Times between 1987 and 2007 [110]. There are approximately 650,000 manually generated article-summary pairs and 1.5 million manually annotated articles. It can be used for automatic summarization, text classification, content extraction, and other NLP tasks. The statistics of the *NYT* dataset are shown in Table 3.

In 2018, Paulus et al. [77] performed a series of preprocessing on the original *NYT* dataset to make it suitable for text summarization tasks. After limiting the length of the input document to 800 tokens and summary to 100 tokens, the average length of the document-summary pairs output by their preprocessing steps is 549 tokens for documents and 40 tokens for summaries. Compared with the *CNN/DailyMail* dataset, the summaries of the *NYT* dataset are more varied, shorter, and can utilize higher levels of abstraction and paraphrase. Therefore, these two datasets can complement each other very well.

### 4.5. Newsroom

The *Newsroom*dataset [111] is a large dataset that can be used to train and evaluate automatic summarization systems. This dataset is released by Connected Experiences Laboratory, which consists of 1.3 million news articles and some other metadata. The articles and summaries were manually written by 38 major news publishers, and these data were obtained from searches and social media from 1998 to 2017. The document-summary pairs in the *Newsroom* dataset are processed through some extractive and abstractive preprocessing strategies.

### 4.6. LCSTS

*LCSTS* dataset [112] is a Chinese short text summarization dataset released by the Intelligent Computing Research Center of Harbin Institute of Technology. The dataset is collected from more than 2 million Chinese short texts published by certified users of the SinaWeibo website, which is a Chinese microblogging website. The *LCSTS* dataset contains 2.4 million training pairs, 10,000 validation pairs, and 1,100 testing pairs. The average length of the input text and reference summaries is 104 and 18, respectively. Specially, the validation set and the testing set increase the score of the correlation between the summaries and the original documents. The higher the score, the higher the correlation, which facilitates the researcher to adjust the use of the dataset according to the characteristics of different tasks.

### 4.7. Others

In addition to some mainstream text summarization corpora, there are also some corpora oriented to specific domain tasks, including: news headline generation dataset *XSum* [90], multi-document summarization dataset *Multi-News* [113], conference summary dataset *AMI* [114], IELTS summary dataset *IELTS* [115], academic paper dataset [116], etc. These datasets play very important roles in promoting the development of automatic summarization, and extending the text summarization technology to more fields.

## 5. Performance Analysis

In this section, we introduce the main evaluation metrics of the ABS, including the automatic evaluation and manual evaluation. Then we use these evaluation metrics to analyze the performance of popular ABS models on commonly used datasets.

### 5.1. Evaluation Metrics

#### 5.1.1. Automatic Evaluation

Because it takes considerable time to manually evaluate the performance of the generated summaries on the entire testing set, many automatic evaluation metrics are proposed, such as BLEU, METEOR, and ROUGE. Among them, ROUGE is an automatic recall-oriented summarization evaluation metric proposed by Lin [117], which is the most widely used metric for evaluating the performance of ABS models. It evaluates the quality of the summarization system by counting the number of basic units overlapping between the reference and the generated summaries. The ROUGE metric has been proven to be an effective measure of the quality of summary and is well correlated with human evaluation. There are mainly three commonly used ROUGE metrics: ROUGE-1 (unigram), ROUGE-2 (bigram), and ROUGE-L (Longest Common Subsequence, LCS). ROUGE can only evaluate the character overlap between the reference and the generated summaries, and does not involve semantic evaluation. The calculation is as follows:

(10) $$\begin{array}{c} R_{\text{ROUGE}-N}=\frac{\sum_{S\in \left\{Reference\right\}}\sum_{N_{n-gram}\in S}Count_{match}\left(N_{n-gram}\right)}{\sum_{S\in \left\{Reference\right\}}\sum_{N_{n-gram}\in S}Count\left(N_{n-gram}\right)}, \end{array}$$

where {*Reference*} denotes the reference summaries, *Count*_*match*_(*N*_*n*−*gram*_) denotes the number of *n-grams* in the reference summary and the generated summary at the same time, and *Count*(*N*_*n*−*gram*_) denotes the number of n-grams in the reference summary.

#### 5.1.2. Human Evaluation

The main limitation of the ROUGE metric is that it is coherence-insensitive [118]. Current automatic evaluation metrics can only describe the superficial relationship between sentences, and cannot distinguish the quality of summaries by semantics. Therefore, human evaluation makes up for the shortcomings of automatic evaluation methods to some extent. However, human evaluation is affected by some subjective factors, such as mother tongue, education level, language style, etc. To find a balance and ensure the robustness of the evaluation, many ABS systems perform ROUGE evaluation on the entire testing set, and perform the human evaluation on a sampled small testing set.

At present, human evaluation is mainly carried out from the following aspects:

1. *Readability*. It measures how well the summary is fluent and grammatical.
2. *Informativeness*. It measures how well the summary contains the gist of the original input.
3. *Fluency*. It measures how well the summary is consistent with human language habits.
4. *Conciseness*. It measures whether the summary is simple and easy to understand (less redundancy)
5. *Factual correctness*. It indicates whether the facts described in the summary are consistent with the original document, which is the most critical factor affecting the usability of the summary.

Amazon Mechanical Turk (AMT) is the most widely used crowdsourcing platform. To avoid subjective tendencies, these selected participants are usually not told which one is the reference summary and which one is the generated summary.

### 5.2. Performance Comparison of Popular ABS Models

In this section, we report the ROUGE scores of the popular ABS models on the *CNN/DailyMail* dataset and *Gigaword* dataset.  Table 4 shows the results of SOTA models for each year in the past five years (2017-2021) on the *Gigaword* dataset.  Table 5 shows the results of annual SOTA models on the *CNN/DailyMail* dataset. The results on all datasets are consistent overall. Specially, we also report the vocabulary size used by different methods, including the encoding vocabulary size (input) and the decoding vocabulary (output). They control the vocabulary size to improve the training efficiency. For the models in Tables 4 and 5, we report the techniques they employ, as follows:

- PG + Coverage [95]: a pointer generator network that can copy words directly from the original text and can reduce repetitions using a coverage mechanism.
- SEASS [119]: an RNN-based Seq2seq model that selectively encodes important information in the input to enhance summary generation.
- DRGD [120]: a Seq2seq framework, which can generate summaries using the structural information of the input.
- FTSumg [100]: an RNN-based model that encodes factual descriptions in the input to enhance the factual correctness of the generated summaries.
- Transformer [121]: a fully attention-based framework that is also the foundational component of pretrained models.
- Struct + 2Way + Word [122]: a Seq2seq model that can copy key words and relationships from the original text using structure-infused copy mechanisms.
- PG + EntailGen + QuestionGen [123]: a neural model based on multi-task learning, which can utilize question and entailment generation task to enhance the summary generation process.
- CGU [124]: a global encoding framework that utilizes convolutional gated unit to encode global information of the input.
- Reinforced-Topic-ConvS2S [85]: a convolutional Seq2seq model that can integrate topic and textual information to enhance the summary generation process.
- Seq2seq + E2T_cnn [125]: a Seq2seq model that can utilize linked entities to guide the decoding process.
- Re^3 Sum [126]: a extended Seq2seq framework that can utilize candidate templates to generate summaries.
- JointParsing [127]: a novel Seq2seq model consisting of a sequential decoder and a tree-based decoder, which improves the syntactic correctness of the generated summaries.
- Concept pointer + DS [128]: a concept pointer network which expands the types of words that pointers can copy using knowledge-based conceptualizations.
- MASS [129]: a Seq2seq pretrained LM, which improves the feature extraction ability of the model by jointly training the encoder and decoder.
- UniLM [130]: a novel unified pretrained LM that employs a shared transformer layer and adopts specific self-attention masks during decoding.
- BiSET [131]: a neural bidirectional model that uses the input text to generate templates to guide the summary generation process.
- PEGASUS [132]: a novel pretrained LM that improves the representational power of the model by removing/masking important sentences in the input and then regenerating them.
- ERNIE-GEN [133]: a multi-flow Seq2seq pretrained framework that utilizes the infilling generation and noise-aware mechanism to enhance the generation process. There are two models of different scales (ERNIE-GENBASE and ERNIE-GENLARGE).
- ProphetNet [134]: a novel Seq2seq pretrained model that introduces a self-supervised objective and a n-stream self-attention mechanism.
- BART-RXF [135]: a pretrained LM that reduces representation changes during fine-tuning by replacing used adversarial objectives with parameter noise.
- Mask Attention Network [136]: an improved transformer-based framework that introduces a dynamic mask attention network layer and constructs a sequential layered structure.
- Transformer + Wdrop [137]: a transformer-based model that utilizes a word dropout perturbation to perform training.
- Transformer + Rep [137]: a transformer-based model that utilizes a word replacement perturbation to perform training.
- MUPPET BART Large [138]: a pretrained model that adopts a pre-finetuning technique to significantly improve the efficiency and performance of it.
- ROUGESal + Ent RL [139]: a Seq2seq model that adopts a reinforcement learning strategy to improve the quality of generated summaries from different perspectives.
- RNN-ext + abs + RL + rerank [140]: a fast abstractive summarization that can generate a concise summary by selecting salient sentences and rewriting them.
- Bottom-Up [141]: a novel Seq2seq summarization that utilizes a bottom-up attention as a selector to select salient sentences.
- EditNet [143]: a mixed extractive-abstractive model that utilizes an editorial network to generate summary.
- Two-Stage + RL [144]: a novel Seq2seq pretrained framework that employs a two-stage decoder to generate summary.
- BertSumExtAbs [145]: a pretrained model that employs a document-level encoder based on BERT to obtain the semantic information of input document.
- UniLMv2 [147]: a pseudo-masked LM that utilizes the pretrained LM for both autoencoding and partially autoregressive tasks using a novel training procedure.
- BART + R-Drop [148]: a BART model with R-Drop as its training strategy to regularize dropout.
- GLM-XXLarge [149]: a novel pretrained framework that can improve the generalization and adaptability of neural networks to deal with different downstream tasks.

From the results in the table, we can know that the large-scale language model based on pretraining has achieved the current SOTA results. This is expected, because these pretrained models are pretrained on a large-scale external corpus (e.g., Wikipedia) to capture deeper semantic information of natural language. And now, the models based on pretraining almost dominate the list of various NLP tasks. However, the pretraining process requires enormous computing resources and massive data to support it. Most researchers can only use pretrained models to fine-tune for adapting to specific tasks.

Compared with the Seq2Seq baseline, adding pointers and coverage mechanisms can significantly improve the quality of the generated summary. Furthermore, adding internal guidance information can better control the generation process of the ABS systems, such as keywords, key sentences, etc., which allows the model to focus more on the important parts of the document when decoding, thereby enhancing the informativeness of generated summaries. In addition, the introduction of external information into the system can also further enrich the semantic information of the model, thereby ensuring the readability and factual correctness of the generated summary, such as commonsense knowledge graphs. In particular, the introduction of triples improves the factual correctness and the ROUGE score of generated summaries. Compared with the baseline models, the use of reinforcement learning training strategy further enhances the performance of summarization systems.

## 6. Conclusions

Since the automatic text summarization technology was proposed in the late 1950s, it has gradually developed from extractive to abstractive. In recent years, as deep learning technology has matured in the NLP field, abstractive summarization based on deep neural networks has also made rapid development. Automatic text summarization is not only widely used in finance, news, media, and other fields but also plays an important role in information retrieval, public opinion analysis, and content review.

In this paper, we provide a comprehensive overview of currently available abstractive text summarization models. We show the overall framework of the ABS systems based on neural networks, the details of model design, training strategies, and summarize the advantages and disadvantages of these methods. We also introduced some datasets and evaluation metrics that are widely used in the field of text summarization. Finally, we report the performance analysis results of different models on large-scale datasets, which should be helpful for researchers to choose a suitable framework and model according to their own needs. We hope that our work can provide some new perspectives and inspirations for the future research and application of ABS.

With the amount of data becoming more extensive and the attributes of the data becoming more and more abundant, the ABS models based on deep learning have great potential. However, the existing ABS methods have many limitations, which are the future challenges and research directions of the research community. These challenges will help the researchers to identify areas where further research is needed. We discuss several directions worth studying in the future, as follows:

1. *Personalized Summary Generation*. At present, most of the summary models are based on input documents and do not consider the subjective demands of users. A system that can generate personalized summaries according to specific user needs will be very useful in e-commerce and text-based recommendation.
2. *Introduce Richer External Knowledge*. Both models guided by keywords (sentences) and models enhanced by factual triples essentially use knowledge from within the document. However, with the development of knowledge graph technology, a lot of commonsense knowledge can be used to enhance the model and further improve the factual correctness of the generated summaries.
3. *Flexible Stopping Criteria during the Generation Process*. The generation of a summary is an iterative process. At present, almost all methods limit the maximum length of summary in advance to terminate this process. However, in fact, different scenarios and fields, and even different input documents, have different lengths of the summary. For example, the summary of a scientific article is longer than a news article. How to make the system adaptively terminate the iterative process is a significant research direction.
4. *Comprehensive Evaluation Metrics*. Evaluating the quality of generated summary either automatically or manually is a difficult task. Manual evaluation is highly subjective and can only be performed on a small testing set, which is not statistically significant. However, the current automatic evaluation is difficult to consider the semantic level. Therefore, a new comprehensive automatic evaluation metric is essential, which can not only help evaluate the quality of a summary but also support the training process of the ABS system.
5. *Cross-Language or Low-Resource Language Summarization*. Currently, popular public summarization datasets are based on English. Using these publicly available large-scale English datasets to train a cross-language summarization model to generate summaries in low-resource languages is an interesting and meaningful work. This research is still in its infancy and requires more researchers to work together to make a breakthrough [150].

## Figures and Tables

**Table 1:** The main directions of our efforts in this paper.

Gaps/limitations	The way to address in this paper
Comprehensive classification	**From the perspective of methodology**, we classify some popular models in recent years, which is more convenient for readers to **distinguish and select appropriate models**.
Summary of the new methods	The summary of new methods has always been a very **vague problem**. **This paper is driven by application** and classifies new technologies that have appeared in the past three years, which is more **in line with readers' expectations for new technologies**.
Analysis of results (not limited to papers but also competition results and public large-scale models)	To the best of our knowledge, we are the first to systematically present all SOTA results for that year, including **public literature, competition data, and published large-scale pretrained models, at a time granularity of years**.
Summary from an application perspective	The biggest purpose of this article is to help readers better choose a suitable summarization model, so we discuss the **possible hotspots of future research and some limitations from the perspective of application**.

**Table 2:** The statistics of *DUC/TAC* datasets.

Dataset	#Document	Language	#Ground-truth summary	Summary length
DUC 2001	60 × 10	Eng.	3 per cluster	50, 100, 200, 400 tokens
DUC 2002	60 × 10	Eng.	128	10, 50, 100, 200 tokens
DUC 2003	60 × 10, 30 × 25	Eng.	128	200, 400 tokens
DUC 2004	100 × 10	Ara. & Eng.	4 per cluster	100 tokens
DUC 2005	50 × 32	Eng.	4 per cluster	665 bytes
DUC 2006	50 × 25	Eng.	4 per cluster	250 tokens
DUC 2007	25 × 10	Eng.	4 per cluster	250 tokens
TAC 2008	48 × 20	Eng.	4 per cluster	250 tokens
TAC 2009	44 × 20	Eng.	4 per cluster	250 tokens
TAC 2010	46 × 20	Eng.	8 per cluster	100 tokens
TAC 2011	44 × 20	Eng.	8 per cluster	100 tokens

**Table 3:** The statistics of the standard datasets.

Dataset	Lang.	#Train	#Valid.	#Test.	Ave. source length	Ave. target length
Gigaword	Eng.	3,800,000	189,000	1951	31.4	8.3
CNN/Daily Mail	Eng.	287,226	13,368	11,490	780	56
NYT	Eng.	589,284	32,736	32,739	549	40
Newsroom	Eng.	995,041	105,760	105,760	658.6	26.7
LCSTS	Chi.	2,400,591	10,666	1,106	103.7	17.8

**Table 4:** The results of different models on the *Gigaword* dataset. RG-1 denotes the ROUGE-1 score, RG-2 denotes ROUGE-2 score, and RG-L denotes ROUGE-L score.

Year	Method	Gigaword			Vocabulary
		RG-1	RG-2	RG-L	In/out
2017	SEASS [119]	36.15	17.54	33.63	120k/69k
	DRGD [120]	36.27	17.57	33.62	110k/69k
	FTSumg [100]	37.27	17.65	34.24	120k/69k
	**Transformer** [121]	**37.57**	**18.90**	**34.69**	120k/69k
2018	Struct + 2Way + Word [122]	35.47	17.66	33.52	70k/10k
	PG + EntailGen + QuestionGen [123]	35.98	17.76	33.63	110k/69k
	CGU [124]	36.3	18.0	33.8	110k/69k
	Reinforced-topic-ConvS2S [85]	36.92	18.29	34.58	110k/69k
	Seq2seq + *E*2T_cnn [125]	37.04	16.66	34.93	50k/50k
	**Re^3 sum** [126]	**37.04**	**19.03**	**34.46**	110k/69k
2019	JointParsing [127]	36.61	18.85	34.33	110k/69k
	Concept pointer + DS [128]	37.01	17.10	34.87	150k/150k
	MASS [129]	38.73	19.71	35.96	110k/69k
	UniLM [130]	38.90	20.05	36.00	30k/30k
	BiSET [131]	39.11	19.78	36.87	110k/69k
	**PEGASUS** [132]	39.12	19.86	36.24	96k/96k
2020	ERNIE-GENBASE [133]	38.83	20.04	36.20	50k/50k
	ERNIE-GENLARGE [133]	39.25	20.25	36.53	50k/50k
	ProphetNet [134]	39.51	20.42	36.69	110k/69k
	**BART-RXF** [135]	40.45	20.69	36.56	120k/69k
2021	Mask attention network [136]	38.28	19.46	35.46	110k/69k
	Transformer + Wdrop [137]	39.66	20.45	36.59	32k/32k
	Transformer + Rep [137]	39.81	20.40	36.93	32k/32k
	**MUPPET BART large** [138]	40.4	20.54	36.21	120k/69k

The values in bold represent the SOTA model for that year.

**Table 5:** The results of different models on the *CNN/DailyMail* dataset. RG-1 denotes the ROUGE-1 score, RG-2 denotes the ROUGE-2 score, and RG-L denotes the ROUGE-L score.

Year	Method	CNN/Daily Mail			Vocabulary
		RG-1	RG-2	RG-L	In/out
2017	Transformer [121]	39.50	16.06	36.63	150k/50k
	**PG** + **Coverage** [95]	**39.53**	**17.28**	**36.38**	50k/50k
2018	PG + EntailGen + QuestionGen [123]	39.81	17.64	36.54	150k/60k
	ROUGESal + Ent RL [139]	40.43	18.00	37.10	50k/50k
	Li et al. [83]	40.30	18.02	37.36	50k/50k
	RNN-ext + abs + RL + rerank [140]	40.88	17.80	38.54	30k/30k
	Bottom-up [141]	41.69	19.47	37.92	150k/50k
	**DCA** [142]	**41.22**	**18.68**	**38.34**	150k/50k
2019	EditNet [143]	41.42	19.03	38.36	50k/50k
	Two-stage + RL [144]	41.71	19.49	38.79	30k/30k
	BertSumExtAbs [145]	42.13	19.60	39.18	120k/120k
	UniLM [130]	43.08	20.43	40.34	30k/30k
	BART [146]	44.16	21.28	40.90	120k/120k
	**PEGASUS** [132]	**44.17**	**21.47**	**41.11**	96k/96k
2020	ERNIE-GENBASE [133]	42.30	19.92	39.68	50k/50k
	UniLMv2 [147]	43.16	20.42	40.14	31k/31k
	ERNIE-GENLARGE [133]	44.31	21.35	**41.60**	50k/50k
	**BART** + **R3F** [135]	**44.38**	**21.53**	41.17	120k/120k
2021	Mask attention network [136]	40.98	18.29	37.88	50k/50k
	MUPPET BART large [138]	44.45	21.25	41.4	120k/120k
	BART + R-drop [148]	44.51	**21.58**	41.24	120k/120k
	**GLM-XXLarge** [149]	**44.7**	21.4	**41.4**	32k/32k

The values in bold represent the SOTA model for that year.

## Data Availability

All the datasets mentioned in Section 4 are publicly available, as follows: DUC/TAC: https://duc.nist.gov/ CNN/DailyMail: https://github.com/deepmind/rc-data (Anonymous Version); https://github.com/abisee/cnn-dailymail (non-Anonymous Version); Gigaword: https://catalog.ldc.upenn.edu/LDC2003T05; NYT: https://catalog.ldc.upenn.edu/LDC2008T19; Newsroom: https://lil.nlp.cornell.edu/newsroom/; LCSTS: http://icrc.hitsz.edu.cn/Article/show/139.html.
